# A case series of intracranial hypertension in patients with Turner syndrome, with and without growth hormone therapy

**DOI:** 10.1186/1687-9856-2015-S1-P79

**Published:** 2015-04-28

**Authors:** Emily Pascoe, Hughie Tsang, Catherine Dunlop, Gopi Subramanian, Grant Bateman, Elizabeth Nunn, Donald Anderson, Patricia Crock

**Affiliations:** 1 Dept Paediatric Endocrinology and Diabetes, John Hunter Children’s Hospital, Newcastle, NSW, Australia; 2 Paediatric Ophthalmologists, John Hunter Children’s Hospital, Newcastle, NSW, Australia; 3 Dept Paediatric Neurology, John Hunter Children’s Hospital, Newcastle, NSW, Australia; 4 Dept Medical Imaging, John Hunter Children’s Hospital, Newcastle, NSW, Australia

## 

Intracranial Hypertension (IH) is a known side effect of GH therapy [1], but has many other aetiologies. Turner syndrome patients have growth failure related to loss of the SHOX transcription factor and so respond to high dose GH therapy. Turner patients also have a high incidence of middle ear disease that can cause intracranial hypertension via mastoiditis and venous sinus thrombosis. IH has been reported in Turner syndrome with no predisposing cause.

## Aim

To describe a case series of 4 patients with Turner syndrome who developed IH.

## Methods

Turner syndrome patients were identified prospectively from the Paediatric Endocrine Database (PED) in a University teaching hospital. All patients had routine ophthalmological and ENT assessments prior to GH therapy. Ocular Coherence Tomography (OCT) scanning was done if papilloedema found. MRI and MRV scans were performed.

## Results

26 active patients with Turner Syndrome, age range 2 to 20 years, were identified and four noted to have co-existent IH. Case 1, age 15 years was referred by a neurologist with pre-existing IH and had never received GH therapy. No cause was found. Case 2, age 14 years was asymptomatic and papilloedema found on fundoscopy as part of pre-GH ophthalmology assessment. The patient had severe middle ear disease and had a mastoidectomy. CSF pressure > 35 mmHg. MRV showed abnormal venous drainage and GH therapy was not started. Both patients were treated with Diamox. Case 3, age 14 yrs and Case 4, age 10 yrs, commenced GH at 9.2 mg/m^2^/week (having had normal fundi) and developed headaches and papilloedema, which resolved when GH treatment was ceased. Case 3; CSF pressure 20 mmHg and MRV abnormal sagittal sinus flow. In both patients, GH was successfully restarted at low dose with slow upward titration.

## Conclusions

Intracranial hypertension may be seen in Turner syndrome independently from GH therapy. An ophthalmological consultation prior to starting GH therapy is important as some patients have asymptomatic papilloedema. Having significant middle ear disease may add to the risk.
